# Thromboelastography may detect hypercoagulation in early sepsis and improve anticoagulation during extracorporeal treatments

**DOI:** 10.1186/cc14421

**Published:** 2015-03-16

**Authors:** F Turani, S Busatti, R Barchetta, AB Belli, S Martini, M Falco

**Affiliations:** 1 Aurelia and European Hospital, Rome, Italy

## Introduction

During early sepsis, activation of the inflammatory response and coagulation occurs. Extracorporeal therapies are used to adsorb mediators, but the coagulation of filters is a drawback [1,2]. The aim of this study is to evaluate whether thromboelastography (TEG) may detect hypercoagulation and may improve anticoagulation during extracorporeal treatments.

## Methods

Twenty-four patients with early severe sepsis had a TEG monitoring at basal time (T0) and during three different extracorporeal treatments (T1): coupled plasma filtration (CPFA) with heparin infusion (Group A), CPFA with citrate infusion (Group B) and RRT with oXiris filter - heparin coated - and no heparin infusion (Group C). ANOVA test was used for the statistical analysis.

## Results

Table 1 presents the TEG values in early septic patients at T0. At T1, angle and MA decreased and *r *increased in Group A at difference with Group B and Group C (*P *< 0.01). In group C, LY 30 was higher than in Group A and B (*P *< 0.01).

**Table 1:** 

	Control	Sepsis
*r*	8 ± 2	6 ± 3
*k*	4 ± 2	1.7 ± 0.5*
Angle	47 ± 15	67 ± 7*
MA	55 ± 8	72 ± 5*
LYS 30	1.8 ± 1	0.95 ± 0.9

*P < 0.01.

## Conclusion

In early sepsis, TEG monitoring may detect hypercoagulability. CPFA with heparin, but not CPFA with citrate and oXiris, is able to reverse hypercoagulability. OXiris may induce fibrinolysis. TEG detects alterations of coagulation during early sepsis and extracorporeal treatments.
